# Intrachannel delivery of a drainage tube beyond the endoscope tip for continuous low-pressure saline perfusion during endoscopic submucosal dissection

**DOI:** 10.1055/a-2919-1256

**Published:** 2026-08-14

**Authors:** Kosei Hashimoto, Masafumi Kitamura, Takashi Ueno, Satoshi Sato, Yuka Kagaya, Yuji Ino, Hironori Yamamoto

**Affiliations:** 1 Department of Gastroenterology 13882 Tochigi Cancer CenterUtsunomiyaTochigi PrefectureJapan; 2 Department of Medicine, Division of Gastroenterology 12838 Jichi Medical UniversityShimotsukeTochigi PrefectureJapan; 3 Department of Endoscopic Research and International Education Funded by FUJIFILM Medical Co., Ltd. 12838 Jichi Medical UniversityShimotsukeTochigi PrefectureJapan

We have previously reported the effectiveness of the continuous low-pressure saline perfusion (CLPSP) method in endoscopic submucosal dissection (ESD). 1​ ​2​ CLPSP is an effective method that achieves the two essential conditions for safe and efficient ESD: maintaining a consistently collapsed lumen and maximizing the benefits of saline immersion, maintaining a clear view by continuous perfusion.

Regarding drainage techniques, we have reported the use of Foley catheters, alpha-loop-shaped tubes and the scope-wrapping method (Asclepius tube) in colorectal ESD 3​ 4​ ​5​ , as well as clip fixation of the tube tip to the gastric mucosa for gastric ESD. 1​ However, clip fixation to the gastric mucosa can detach due to friction between the tube and the endoscope. Furthermore, these methods have limitations, such as insufficient aspiration of gas and fluid that accumulates beyond the endoscope.

**Fig. 1:**
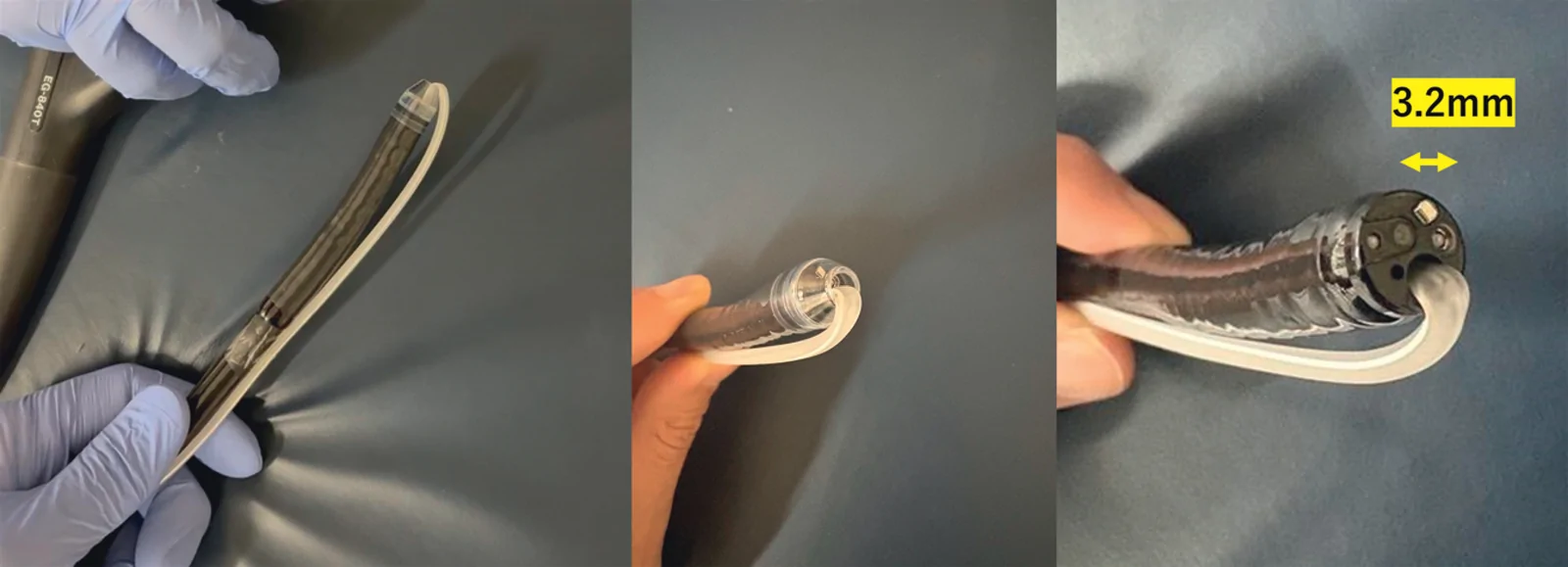
The drainage tube is secured to the scope with parafilm. The tip of the tube is bent to the desired length and inserted into the accessory channel. Because the working channel diameter of a therapeutic endoscope is 3.2 mm, a tube size of ≤9 Fr is required.

To address these limitations, we devised a simple technique: the drainage tube is secured to the scope with parafilm, the tip of the tube is bent to the desired length, inserted into the accessory channel and delivered into the lumen ( Video 1 ). Because the working channel diameter of a therapeutic endoscope is 3.2 mm, a tube size of ≤9 Fr is required ( Fig. 1 ). Insertion of an injection needle through the channel pushes out the tube, allowing the tip to be deployed and positioned distal to the lesion.

**Video 1** A simple and effective delivery beyond the endoscope tip for the continuous low-pressure saline perfusion (CLPSP)-ESD.


**Fig. 2:**
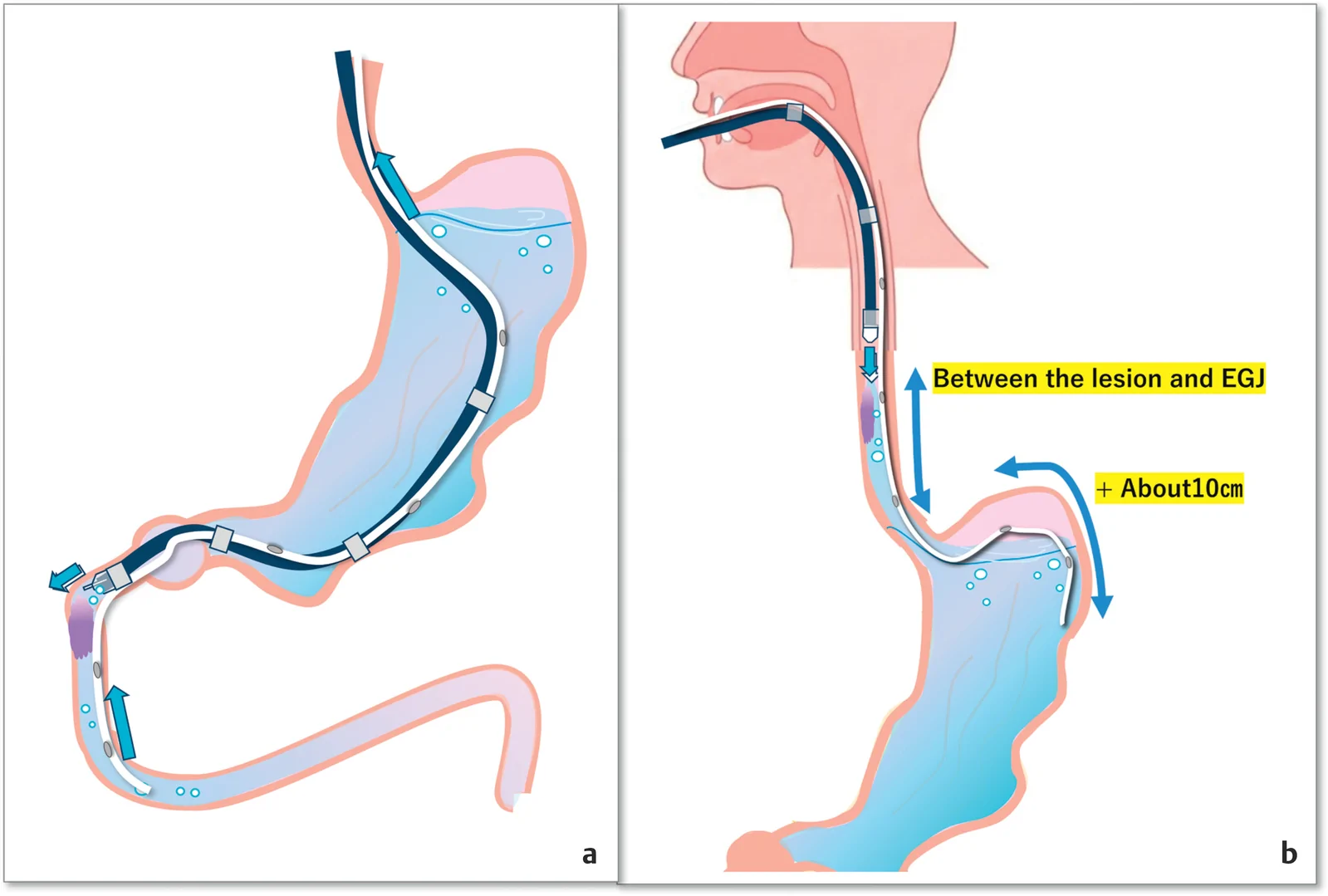
Because the tip of the tube is positioned distal to the lesion, it enables efficient recovery of saline flowing toward the anal side. ( **a** ) Duodenal ESD setting. ( **b** ) Esophageal ESD setting.

In the duodenal ESD, because the tip of the tube is positioned distal to the lesion, it enables efficient recovery of saline flowing toward the anal side, thereby reducing the risk of saline overload ( Fig. 2a ). In esophageal ESD, adjusting the folded length of the tube enables the tip to reach the stomach, facilitating continuous drainage of both esophageal and gastric gas and fluid ( Fig. 2b ). This technique is also applicable to gastric and colorectal ESD.

This method represents a very simple, effective, and cost-efficient drainage strategy for CLPSP-ESD.

Endoscopy_UCTN_Code_TTT_1AQ_2AD_3AD
